# Postoperative Monacolin K Supplementation and Lipid Profile After Sleeve Gastrectomy: A Retrospective Comparative Analysis in Women

**DOI:** 10.3390/nu18040647

**Published:** 2026-02-16

**Authors:** Krzysztof Nocoń, Urszula Kukla, Daria Gendosz de Carrillo, Claudia Wawrzynosek, Halina Jędrzejowska-Szypułka, Dominika Krakowczyk, Aleksander J. Owczarek, Kamila Szeliga, Tomasz Sawczyn

**Affiliations:** 1 Department of General, Endocrine and Oncological Surgery, Multi-Specialty Hospital in Jaworzno, Józefa Chełmońskiego 28 Street, 43-600 Jaworzno, Poland; krzysztof.nocon@szpital.jaworzno.pl (K.N.); urszula.kukla@szpital.jaworzno.pl (U.K.); 2 Department of Physiology, Faculty of Medical Sciences in Katowice, Medical University of Silesia in Katowice, Medyków 18 Street, 40-752 Katowice, Poland; dgendosz@sum.edu.pl (D.G.d.C.); claudia.waldner02@gmail.com (C.W.); hszypulka@sum.edu.pl (H.J.-S.); 3 Saint John Paul II Upper Silesian Child Health Centre, Public Clinical Hospital No. 6 of the Medical University of Silesia in Katowice, Medyków 16 Street, 40-752 Katowice, Poland; dominika.krakowczyk@sum.edu.pl; 4 Health Promotion and Obesity Management Unit, Department of Pathophysiology, Faculty of Medical Sciences in Katowice, Medical University of Silesia in Katowice, Medyków 18 Street, 40-752 Katowice, Poland; aowczarek@sum.edu.pl; 5 Department of Pediatric and Pediatric Endocrinology, Faculty of Medical Sciences in Katowice, Medical University of Silesia in Katowice, Medyków 16 Street, 40-752 Katowice, Poland; kszeliga@sum.edu.pl

**Keywords:** sleeve gastrectomy, monacolin K, bariatric surgery, metabolic surgery, lipid profile, obesity

## Abstract

**Background:** Sleeve gastrectomy (SG) reliably reduces weight and triglycerides, but LDL-C responses are variable. In this retrospective observational study, we evaluated whether adjunctive monacolin K (red yeast rice; 3 mg/day) improves early lipid modulation after SG. **Methods:** In this single-center retrospective study of women only, 149 patients undergoing SG within the national KOS-BAR program were analyzed in four groups: controls without supplementation (CG, n = 62) and three supplementation cohorts receiving monacolin K for 6 months (G1 early (from week 1; n = 46), G2 delayed (months 3–9; n = 10), and G3 delayed (months 6–12; n = 31)). Outcomes included total cholesterol (TC), LDL-C, HDL, and triglycerides (TG). Missing data were imputed; mixed models for repeated measures assessed longitudinal changes. **Results:** From baseline to 6 months, LDL-C-C increased in the control group (CG; +21.9 mg/dL) and decreased in G1 (mean change: −11.1 mg/dL), with a significant group-by-time interaction (*p* < 0.001). HDL-C increased in both CG and G1, whereas triglyceride levels decreased more markedly in G1 than in CG (−36.2 vs. −19.6 mg/dL). Total cholesterol decreased in G1 (−13.4 mg/dL) and in G2 at 9 months (−22.5 mg/dL). **Conclusions:** In the early supplementation group, LDL-C-C levels decreased over the first 6 months after SG, whereas an increase was observed in the control group, which had significantly lower baseline LDL-C concentrations. In women undergoing SG, early postoperative monacolin K supplementation was associated with LDL-C stabilization and enhanced lipid optimization without impeding weight-loss benefits. Delayed initiation yields partial improvements, especially for TG and HDL-C. These observations underscore the need for prospective, sex-stratified studies with appropriate baseline adjustments to clarify the association between monacolin K use and postoperative lipid trajectories after SG.

## 1. Introduction

Obesity is a chronic, progressive disease and a major global health challenge, which is strongly associated with dyslipidemia, hypertension, type 2 diabetes mellitus (T2DM), and increased cardiovascular risk [1]. Bariatric surgery provides sustained weight loss and remission of multiple obesity-related comorbidities, including dyslipidemia, with a reported 67% reduction in its prevalence within 2–5 years after surgery [1,2,3,4]. Sleeve gastrectomy (SG) is currently the most frequently performed bariatric procedure worldwide, accounting for approximately 63.3% of all bariatric operations [5]. Although SG consistently increases high-density lipoprotein cholesterol (HDL-C) and reduces triglycerides (TG), its effects on total cholesterol (TC) and low-density lipoprotein cholesterol (LDL-C) remain variable [2,3,4,5]. Previous reports suggest that some lipid improvements after SG may occur independently of age and weight loss and have been observed in both sexes; however, sex differences in lipid metabolism may still be clinically relevant [6,7,8]. 

Red yeast rice contains monacolin K, a natural inhibitor of 3-hydroxy-3-methylglutaryl coenzyme A reductase that is chemically identical to lovastatin [9]. Monacolin K effectively lowers LDL-C in individuals with mild to moderate hypercholesterolemia [10,11] and has also been shown to reduce TC, apolipoprotein B, and blood pressure, contributing to decreased cardiovascular risk [12]. However, its use remains controversial due to statin-like adverse effect potential and variability in nutraceutical formulations, leading regulatory authorities, including the European Food Safety Authority, to question safe intake thresholds [13]. At the time this study was conducted, monacolin K (3 mg/day) was widely used as a lipid-lowering nutraceutical, and adverse event reporting systems indicated a very low proportion of muscle-related and hepatobiliary events (<0.01%), consistent with meta-analyses showing no increased risk of myopathy or liver dysfunction [14]. Subsequent regulatory changes occurred only after completion of patient recruitment and follow-up and therefore did not influence clinical decision-making in this cohort.

While SG alone typically improves HDL-C and TG, LDL-C reduction is inconsistent [2,3,4,5]. In contrast, monacolin K has demonstrated consistent LDL-C–lowering effects across clinical studies [10,13]. We therefore hypothesized that postoperative monacolin K supplementation might enhance lipid profile optimization after SG.

The objective of this retrospective study was to evaluate the association between postoperative monacolin K supplementation and lipid profile changes in women undergoing SG, and to explore whether the timing of supplementation initiation influences lipid trajectories. We focused exclusively on female patients to ensure cohort homogeneity and minimize variability related to well-established sex-specific differences in lipid metabolism [15,16].

Importantly, this study provides real-world longitudinal evidence on lipid trajectories after sleeve gastrectomy by comparing early versus delayed initiation of monacolin K supplementation. By focusing on LDL-C dynamics, which remain inconsistent after SG alone, our analysis addresses a clinically relevant gap regarding postoperative lipid management. In addition to evaluating early supplementation, we also explored delayed initiation in routine follow-up, offering insight into pragmatic treatment pathways encountered in everyday bariatric practice.

## 2. Materials and Methods

This retrospective study included female patients who underwent SG in 2022–2024 as part of the Comprehensive Medical Care Program for Patients with Morbid Obesity Treated Surgically (KOS-BAR), coordinated by the Polish Ministry of Health. All surgical procedures were performed at the Multispecialty Hospital in Jaworzno. Participants were qualified for surgery in accordance with the national eligibility criteria, which included a body mass index (BMI) of ≥40 kg/m^2^ or ≥35 kg/m^2^ in the presence of at least one obesity-related associated medical problems such as type 2 diabetes, arterial hypertension, dyslipidemia, or obstructive sleep apnea. Patient enrollment and care were conducted within the framework of this publicly funded national program aimed at improving long-term outcomes in the treatment of severe obesity. Male patients constituted approximately 2% of the KOS-BAR cohort, reflecting low male participation in this publicly funded bariatric program; the well-established sex-related differences in lipid metabolism further justified their exclusion. Men generally present with higher LDL-C concentrations in early and middle adulthood, whereas in women, the menopausal transition is associated with a shift toward a more atherogenic lipid profile [17,18]. Therefore, restricting the analysis to women allowed for more consistent and reliable within-group comparisons. Additionally, participants with conditions known to affect lipid metabolism, such as chronic kidney disease, hypothyroidism, and other secondary causes of dyslipidemia, were excluded to minimize confounding effects. The study was reported in accordance with the STROBE guidelines for observational studies.

## 3. Study Groups and Supplementation Protocol

**Figure 1:**
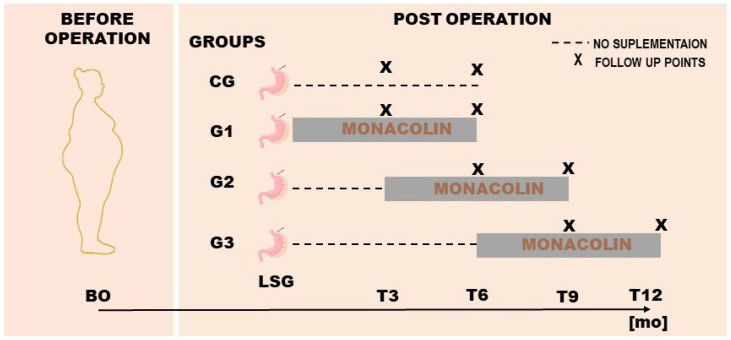
Study groups were defined by the timing of monacolin K supplementation after SG. Legends: CG—no supplementation; G1—postoperative supplementation from week 1; G2—postoperative supplementation from month 3 to 9; G3—postoperative supplementation from month 6 to 12. All supplemented groups received monacolin K 3 mg/day for 6 months; X—indicates follow-up time points.

In this retrospective analysis, patients were categorized into subgroups based on whether and when they initiated monacolin K supplementation following SG. All supplemented patients received the same commercially available preparation providing 3 mg/day of monacolin K. Monacolin K supplementation was initiated during routine clinical care at the discretion of the treating physician, primarily in patients with persistently elevated LDL-C levels or insufficient lipid improvement after sleeve gastrectomy. Adherence was assessed during routine outpatient visits based on patient self-report; pill counts or biochemical verification were not available in this retrospective analysis. The control group (CG) included patients who did not receive monacolin K at any stage postoperatively and underwent follow-up assessments at 3 and 6 months after surgery (Figure 1).

Among those who received monacolin K, three intervention groups were defined according to the timing of supplementation initiation but with a consistent duration of use (6 months). The G1 group (n = 46) began supplementation within the first postoperative week and was assessed at 6 months, and served as the primary comparison group against the control group. The G2 group (n = 10) initiated supplementation between months 3 and 9 postoperatively, and the G3 group (n = 31) started between months 6 and 12 (Figure 1). This stratification allowed for analysis not only of the combined effects of SG and monacolin K, but also the potential influence of supplementation timing on lipid profile outcomes.

Baseline characteristics for the intervention groups are summarized in Table 1. The mean age was comparable across G1, G2, and G3, indicating no significant differences in age distribution. The prevalence of obesity-related comorbidities ranged from 58.1% in G3 to 90.0% in G2. While the between-group difference was not statistically significant (*p* = 0.06), the result approaches the threshold for significance and may reflect clinically meaningful disparities in baseline metabolic burden. Median baseline BMI values were also similar across groups, ranging from 39.8 to 43.3 kg/m^2^.

### 3.1. Surgical Procedure: SG

All patients underwent SG performed by experienced bariatric surgeons using the standardized technique [19]. The procedure consisted of a longitudinal resection of the greater curvature of the stomach, beginning approximately 5 cm proximal to the pylorus and extending up to the angle of His. The fundus was meticulously mobilized to ensure complete dissection from the surrounding structures, including the left diaphragmatic crus.

A French 36 bougie was introduced orally and positioned along the lesser curvature to calibrate the newly created gastric sleeve. Gastric transection was achieved using a linear stapling device in multiple firings, with reinforcement of the staple line applied when indicated. The resected stomach was retrieved through the enlarged port site using a laparoscopic specimen bag.

To ensure the integrity of the staple line, an intraoperative leak test with methylene blue was routinely performed. Trocar sites larger than 5 mm were closed at the fascial level, and skin incisions were approximated with absorbable sutures.

All procedures were conducted under general anesthesia following standard perioperative protocols. Patient selection adhered to Polish Recommendations for bariatric and metabolic surgery.

### 3.2. Nutritional Care

All patients were managed within a standardized perioperative nutritional protocol as part of the KOS-BAR program. Preoperative preparation included education on thorough chewing, appropriate hydration between meals, avoidance of carbonated beverages, and routine micronutrient supplementation, including bariatric-specific multivitamins and zinc. Postoperatively, patients were instructed to consume regular meals (at least five per day), eat slowly (minimum 20 min per meal), avoid alcohol and snacking between meals, and stop eating at satiety. Caloric targets were staged: 700–800 kcal/day during the first postoperative month (optimal ~1000 kcal), ≥1100–1200 kcal/day by the end of the second month, and ≥1300 kcal/day from the third month onward (typically 1300–2000 kcal/day depending on individual requirements). Recommended macronutrient targets included approximately 100 g/day of carbohydrates, protein intake of at least 60 g/day in women and 80 g/day in men (optimal ~1.4 g/kg ideal body weight), and fat intake of 50–60 g/day, with an approximate macronutrient distribution of 30% fat, 40–45% carbohydrates, and 25% protein. Patients had unrestricted access to dietetic consultations throughout follow-up; however, quantitative dietary intake and physical activity were not systematically recorded.

### 3.3. Safety Monitoring

Paired ALT and AST measurements before initiation and after approximately 6 months of monacolin K supplementation in the early supplementation group (G1) were collected as part of routine safety monitoring to ensure clinical oversight during supplementation and are summarized in Supplementary Table S1. Importantly, all supplementation groups received monacolin K for an identical duration of 6 months, differing only in the timing of initiation.

### 3.4. Biochemical Analyses

All biochemical analyses were performed in a certified clinical laboratory using standardized enzymatic methods.

## 4. Statistical Analysis

Statistical analysis was performed using R software (v 4.4.0; 2024-04-24 urct, “Puppy Cup”, R Development Core Team (2008), R: A language and environment for statistical computing, R Foundation for Statistical Computing, Vienna, Austria). Statistical significance was set at a *p*-value below 0.05. All tests were two-tailed. The multivariate imputation by chained equation with multiple imputation by distance-aided donor selection for interval data (MICE) was used to impute missing data. To impute univariate missing data, the predictive mean matching method was used (‘midastouch’ method). Imputations were performed using packages ‘mice’ and ‘futuremice’, allowing for parallel computing based on 512 imputed datasets with 5 iterations in the predictive mean matching calculation. The proportion of missing lipid measurements was approximately 35% across follow-up time points, with comparable completeness between the supplementation groups (G1: 27.2%, G2: 25.6%, G3: 26.6%) and slightly, but not significantly higher missingness in the control group (31.5%). On all imputed datasets, a proper longitudinal analysis model was used (linear mixed-effect models for repeated measures–package ‘mmrm’; with contrasts as a post hoc analysis), with baseline lipid values included as covariates in all models, and the results were pooled (with Rubin’s rules). Nominal and ordinal data were expressed as percentages, while interval data were expressed as mean value ± standard deviation in the case of a normal distribution, and as median with lower and upper quartiles otherwise. For imputed data, mean values with 95% confidence intervals (CIs), calculated with the package ‘emmeans’, were presented. Categorical variables were compared using the χ^2^ test. Multiple comparisons were corrected with the Hochberg method. Pooled linear regression was used to assess differences between groups in the raw values as well as the relative change between the last and baseline value for each analyzed variable. No formal sample size calculation was performed due to the retrospective design.

## 5. Results

In this retrospective part of the study, the results section is structured in two parts: the first presents a comparison of patients who initiated early postoperative monacolin K supplementation (G1) versus those who did not receive supplementation (control group (CG)), with measurements taken at baseline before the operation (BO), and three months (T3) and six months (T6) after operation, all reported with 95% CIs. The second part addresses delayed supplementation, focusing on groups G2 and G3, where analyses evaluate changes only within the supplemented groups between BO and the final follow-up, without further reference to the control group.

### 5.1. Early Monacolin K Supplementation Post-SG: Comparative Analysis with Non-Supplemented Controls

#### 5.1.1. Total Cholesterol (TC)

**Figure 2:**
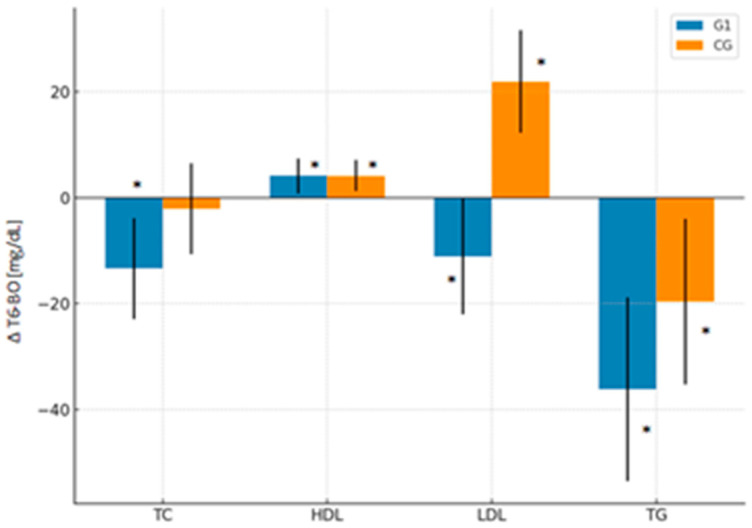
Absolute changes (Δ_T6–BO_) in lipid parameters (mean ± 95% CI) in women undergoing SG with early postoperative monacolin K supplementation (G1) compared to the control group without supplementation (CG). Bars represent change between baseline (BO) and 6 months postoperatively (T6). * indicates significant differences between T6 and BO (*p* < 0.05).

**Figure 3:**
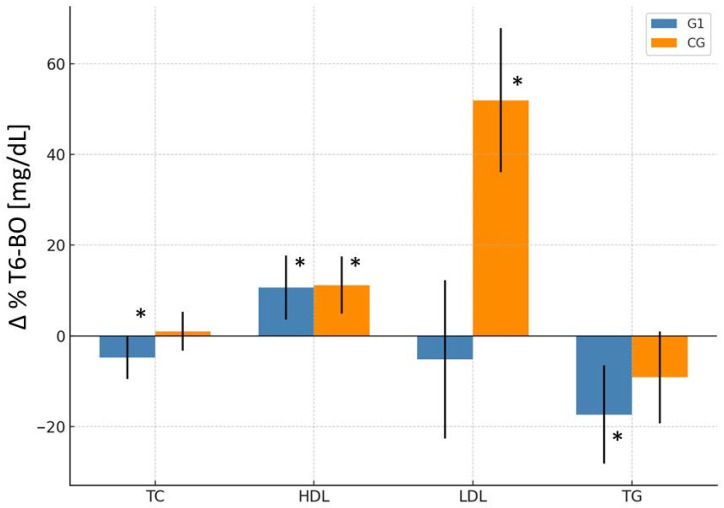
Percentage changes (Δ_T6–BO_) in lipid parameters (mean ± 95% CI) in women undergoing SG with early postoperative monacolin K supplementation (G1) compared to the control group without supplementation (CG). Bars represent change between baseline (BO) and 6 months postoperatively (T6). * indicates significant differences between T6 and BO (*p* < 0.05).

In G1, the mean total cholesterol value decreased significantly from 213.1 mg/dL at BO to 199.7 mg/dL at T6 (*p* < 0.01), representing an absolute reduction of −13.4 mg/dL (95% CI: −22.9 to −3.9) and a relative change of −4.8% (95% CI: −9.5 to −0.1%). In contrast, CG did not show significant changes over time (Δ = −2.1 mg/dL; 95% CI: −10.7 to 6.5; Δ% = +1.0%; 95% CI: −3.3 to 5.3%). The time-group interaction did not reach significance (Table 2; Figure 2 and Figure 3).

#### 5.1.2. LDL-C

LDL-C levels in the group G1 decreased significantly (*p* < 0.05) from 131.7 mg/dL at BO to 120.6 mg/dL at 6 months postoperatively (T6), representing an absolute reduction of 11.1 mg/dL (95% CI: −22.0 to −0.2) and a relative decrease of −5.2% (95% CI: −22.7 to 12.3%). In contrast, CG showed a significant increase from 99.9 mg/dL to 121.8 mg/dL over the same period, an absolute change of 21.9 mg/dL (95% CI: 12.2 to 31.6) and a relative increase of 52.0% (95% CI: 36.1 to 67.9%). A significant group-by-time interaction (*p* < 0.001) was observed: LDL-C levels declined in G1 and increased in CG (Table 2; Figure 2 and Figure 3).

#### 5.1.3. HDL-C

HDL-C increased significantly in both groups at T6. In G1, HDL-C rose from 51.4 to 55.5 mg/dL (*p* < 0.05), while in CG, it increased from 53.1 to 57.2 mg/dL (*p* < 0.001). The relative increase was 10.7% (95% CI: 3.6 to 17.7%) in the monacolin K group and 11.2% (95% CI: 4.9 to 17.5%) in CG. Despite similar magnitudes of improvement, a significant group-by-time interaction was observed (*p* < 0.01), indicating that the temporal pattern of HDL-C changes differed between the groups (Table 2; Figure 2 and Figure 3).

#### 5.1.4. Triglycerides (TG)

TG markedly decreased in both groups. G1 exhibited a more pronounced reduction from 151.9 to 115.7 mg/dL (*p* < 0.001), representing a −36.2 mg/dL (95% CI: −53.5 to −18.8) absolute and −17.4% (95% CI: −28.2 to −6.5%) relative decrease. CG also showed a significant but smaller reduction, from 148.1 to 128.4 mg/dL (Δ = −19.6 mg/dL; 95% CI: −35.3 to −4.0; Δ% = −9.1%; 95% CI: −19.3 to 1.0%). However, no significant group-by-time interaction was observed (*p* = 0.41) (Table 2; Figure 2 and Figure 3).

### 5.2. Delayed Monacolin K Supplementation Post-SG: Effects on Lipid Profile

This part of the study evaluated the effects of delayed initiation of monacolin K supplementation following SG. In G2, supplementation began at 3 months post-operation (T3) and continued until month 9 (T9), while in G3, it was introduced at 6 months post-operation (T6) and continued until month 12 (T12). Measurements were analyzed at baseline, before the operation (BO), and at two respective follow-up points: T6 and T9 for G2; T9 and T12 for G3.

#### 5.2.1. Total Cholesterol After Delayed Supplementation

**Figure 4:**
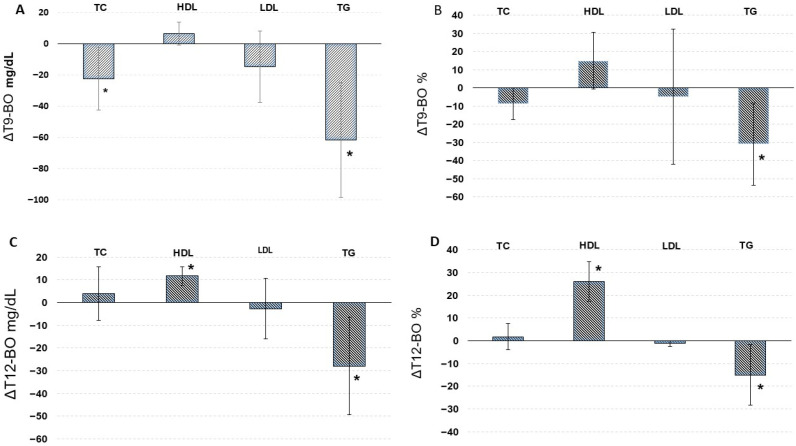
Changes in lipid parameters in women undergoing SG with delayed monacolin K supplementation. (**A**) Absolute changes in group G2 (ΔT9–BO). (**B**) Percentage changes in group G2 (ΔT9–BO). (**C**) Absolute changes in group G3 (ΔT12–BO). (**D**) Percentage changes in group G3 (ΔT12–BO). (mean ± 95% CI). * indicates significance differences between second follow-up and BO (T9 for G2, T12 for G3) (*p* < 0.05).

In G2, total cholesterol decreased from 214.9 mg/dL at baseline to 192.4 mg/dL at T9, with the reduction reaching statistical significance (*p* < 0.05). This corresponds to an absolute change of −22.5 mg/dL (95% CI: −42.4 to −2.6) and a relative reduction of −8.8% (95% CI: −18.6 to 0.9%). In G3, total cholesterol remained stable throughout the observation period, with a non-significant numerical increase from 210.9 mg/dL at baseline to 214.8 mg/dL at T12 (Δ = +3.9 mg/dL; 95% CI: −7.8 to 15.6; Δ% = +1.8%; 95% CI: −3.8 to 7.6%). While the overall effect of time was statistically significant (*p* < 0.05), neither the between-group effect (*p* = 0.54) nor the group-by-time interaction (*p* = 0.16) reached significance (Table 2; Figure 4)

#### 5.2.2. HDL-C After Delayed Supplementation

Both groups experienced improvement in HDL-C levels over the supplementation period. In G2, HDL-C increased from 53.3 to 59.7 mg/dL, a relative increase of 14.9% (95% CI: -0.8 to 30.6%), although this change did not reach statistical significance. In contrast, G3 showed a highly significant increase from 53.0 to 64.7 mg/dL (*p* < 0.001), a Δ% of 26.1% (95% CI: 17.5 to 34.7%). A highly significant group-by-time interaction was observed (*p* < 0.01) (Table 2; Figure 4).

#### 5.2.3. LDL-C After Delayed Supplementation

LDL-C levels decreased over time in G2 from 128.9 to 114.1 mg/dL, an absolute change of −14.8 mg/dL (95% CI: −37.7 to 8.1) and a relative change of −4.9% (95% CI: −42.1 to 32.3%), though this reduction did not reach statistical significance. In G3, LDL-C levels remained relatively stable, with only a minor, non-significant change from baseline (Δ = −2.7 mg/dL; 95% CI: −16.0 to 10.5). Nevertheless, a highly significant group-by-time interaction was observed (*p* < 0.001), indicating that the temporal pattern of LDL-C changes differed between the groups (Table 2; Figure 4).

#### 5.2.4. Triglycerides (TG) After Delayed Supplementation

Both G2 and G3 experienced significant reductions in triglyceride levels following the initiation of supplementation. In G2, TG levels decreased from 164.1 to 102.5 mg/dL (*p* < 0.01), an absolute change of −61.6 mg/dL (95% CI: −98.3 to −24.9) and a relative decrease of −30.9% (95% CI: −53.6 to −8.2%). Similarly, G3 demonstrated a significant reduction from 146.8 to 118.8 mg/dL (*p* < 0.05), a relative decrease of −15.1% (95% CI: −28.4 to −1.7%). A significant time effect was observed (*p* < 0.001), though no group or interaction effects reached statistical significance (Table 2; Figure 4).

### 5.3. Body Mass Reduction Following SG

**Figure 5:**
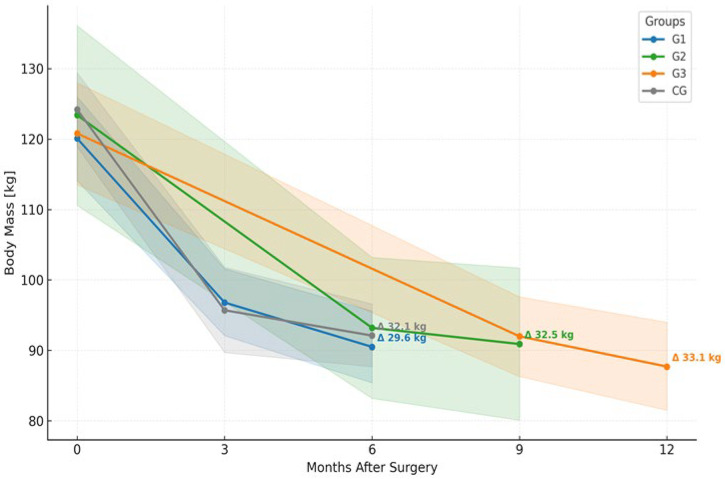
Changes in body mass over time in study groups following SG. Mean body weight trajectories are presented for patients supplemented with monacolin K (G1, G2, G3) and CG without supplementation. Follow-up time points differ between groups according to the supplementation protocol: G1 (BO, 3 and 6 months), G2 (BO, 6 and 9 months), G3 (BO, 9 and 12 months), and CG (BO, 3 and 6 months). Data are presented as means with 95% confidence intervals (shaded areas). Significant and consistent reductions in body mass were observed in all groups over time (*p* < 0.001), with no significant differences between groups (*p* = 0.77) or group-by-time interactions (*p* = 0.73), indicating that weight loss was primarily driven by the surgical intervention.

All study groups demonstrated substantial and statistically significant reductions in body mass following SG. In CG, the mean weight decreased from 124.2 kg at baseline to 92.1 kg at 6 months postoperatively (*p* < 0.001) (Figure 5), corresponding to an absolute reduction of −32.1 kg (95% CI: −365 to −27.6). This reduction was comparable in magnitude to those observed in the intervention groups, and no significant differences were found between groups (*p* = 0.77). In G1, body mass decreased from 120.1 kg at baseline to 90.5 kg at 6 months postoperatively (*p* < 0.001) (Figure 5), corresponding to an absolute reduction of −29.6 kg (95% CI: −33.8 to −25.4) and a relative change of −24.5% (95% CI: −27.2 to −21.8%). In G2, body mass declined from 123.4 kg to 90.9 kg at 9 months (*p* < 0.001) (Figure 5), an absolute loss of −32.5 kg (95% CI: −41.3 to −23.6) and a relative reduction of -26.4% (95% CI: −31.9 to −20.8%). Similarly, G3 showed a decrease from 120.8 kg to 87.7 kg at 12 months (*p* < 0.001) (Figure 5), reflecting an absolute change of −33.0 kg (95% CI: −38.2 to −27.8) and a relative decrease of −27.2% (95% CI: −30.6 to −23.9%). A highly significant effect of time was observed in all groups (*p* < 0.001), while no significant group effect (*p* = 0.77) or group-by-time interaction (*p* = 0.73) was detected, indicating consistent weight loss dynamics regardless of group allocation. The data presented in Table 3 indicate that %EWL and %TBWL values were similar across all study groups, with no statistically significant differences observed (*p* > 0.05).

## 6. Discussion

Sleeve gastrectomy (SG) has become the predominant bariatric procedure worldwide due to favorable perioperative outcomes, procedural feasibility, and an improved safety profile. Since 2018, SG has surpassed Roux-en-Y gastric bypass in procedural frequency and currently accounts for approximately 63.3% of bariatric operations globally [20,21].

Given the growing evidence of procedure-specific effects on lipid metabolism, particularly LDL-C, our findings contribute to the discussion on whether baseline lipid profiles should influence surgical decision-making. In the present study, LDL-C increased significantly at 6 months after SG in the control group, whereas early postoperative monacolin K supplementation prevented this rise. HDL-C increased in both groups, although with differing temporal patterns. These observations suggest an association between early monacolin K use and postoperative lipid trajectories; however, the interpretation is limited by the retrospective design, baseline LDL-C imbalance, and potential selection bias. Importantly, SG alone may have limited or inconsistent effects on LDL-C, a finding supported by prior studies showing minimal or transient LDL-C reductions after SG compared with malabsorptive procedures [2,3,4,5,6,7].

Mechanistic data from randomized studies indicate that SG increases intestinal cholesterol absorption markers (sitosterol, campesterol, cholestanol), whereas RYGB reduces or stabilizes them, while endogenous cholesterol synthesis decreases after both procedures [6]. These findings suggest that postoperative LDL-C changes are primarily driven by altered absorption rather than synthesis. Although absorption and synthesis markers were not measured in our study, the LDL-C increase observed in non-supplemented patients is consistent with reports demonstrating enhanced cholesterol absorption after SG [6]. Monacolin K inhibits HMG-CoA reductase and reduces cholesterol synthesis, reflected by decreased desmosterol and lathosterol levels, while leaving absorption markers largely unchanged [22]. We therefore hypothesize that monacolin K may counterbalance increased intestinal cholesterol uptake after SG. These mechanistic interpretations remain speculative and require confirmation in prospective studies incorporating dedicated biomarkers.

Our cohort only included women as male patients represented approximately 2% of the KOS-BAR program. This disproportionate enrollment may reflect sex-specific health-seeking behaviors and limits generalization to men. At the same time, restricting the analysis to women reduced biological heterogeneity. Sex and menopause-related differences in lipid metabolism are well established, with menopause associated with adverse lipid changes [15,16,17,18]. Although menopausal status was not systematically assessed, our age range likely spanned pre-, peri- and postmenopause and monacolin K consistently stabilized LDL-C across the cohort. Nonetheless, menopause-stratified prospective studies are warranted.

HDL-C increased significantly in both the supplemented and control groups, consistent with previous reports showing progressive HDL-C elevation after SG regardless of supplementation [23]. Total cholesterol only decreased in the supplemented group, suggesting that HDL-C improvement is primarily surgery-driven, whereas TC reduction may be enhanced by monacolin K. Triglycerides decreased significantly in both groups, with a more pronounced reduction in G1, in line with evidence that SG lowers TG mainly through rapid weight loss and improved insulin sensitivity [3,23]. Together, these findings indicate that SG induces favorable lipid changes, while monacolin K may provide additive benefits, particularly for total cholesterol.

Marked improvements in glycemic control were observed across all (Figure S1 Supplement), consistent with prior studies demonstrating reductions in fasting glucose and HbA1c after SG driven mainly by weight loss and enhanced insulin sensitivity [24,25]. All patients received structured nutritional counseling, including individualized dietary planning and long-term follow-up, which is recognized as essential for sustaining metabolic benefits after bariatric surgery [26,27].

Early postoperative monacolin K supplementation was associated with the most robust lipid effects. Delayed initiation also appeared beneficial in selected patients with persistent dyslipidemia, although the responses were less consistent. In G2, LDL-C decreased modestly without reaching statistical significance, while G3 showed largely stable LDL-C levels. Despite these heterogeneous patterns, a significant group-by-time interaction indicates that lipid trajectories differed according to supplementation timing. These subgroup analyses should be considered exploratory due to the limited sample sizes.

Triglyceride reductions were substantial in both delayed groups, although attenuation at 12 months likely reflects the postoperative weight-loss plateau, metabolic adaptation, and declining adherence to dietary recommendations, phenomena commonly observed between 6 and 12 months after SG [28,29,30]. Improvements in fasting glucose and HbA1c similarly plateaued after early postoperative gains, consistent with previous reports [24,31].

All groups experienced significant weight loss during the first 6 months, followed by stabilization up to 12 months [28]. The observed metabolic improvements are largely attributable to postoperative weight reduction and enhanced insulin sensitivity [32,33,34]. Our findings suggest that monacolin K may offer additional support for LDL-C stabilization and HDL-C improvement during the weight-stable phase [35]. Given evolving regulatory assessments and the absence of guideline-based recommendations, monacolin K should not be considered standard therapy but rather a nutraceutical used by some individuals with hypercholesterolemia. Overall, while SG remains the primary driver of metabolic change, adjunctive monacolin K supplementation may help optimize lipid profiles in selected female patients.

## 7. Limitations of the Study

This study has several limitations. Its retrospective observational design precludes causal inference and is inherently susceptible to selection bias as monacolin K initiation was based on routine clinical judgment rather than randomization. Consequently, confounding by indication and regression to the mean cannot be excluded, despite adjustment for baseline lipid values.

The analysis was restricted to female patients, improving cohort homogeneity but limiting generalizability to male populations and not accounting for potential menopausal effects. Delayed supplementation groups (G2 and G3) were small and heterogeneous, rendering these findings exploratory and underpowered.

Although postoperative dietary counseling is standardized and patients had open access to dietetic consultations, quantitative dietary intake, physical activity, and use of additional supplements were not systematically captured, representing residual confounding factors.

Safety assessment relied on routine postoperative laboratory testing and clinical documentation. Paired ALT/AST data were only available for the early supplementation group, and creatine kinase was not systematically monitored. While no clinically relevant adverse effects were observed, comprehensive statin-like safety surveillance was not performed. In addition, monacolin K has statin-like activity, and its risk assessment is complicated by variability in nutraceutical content: analyses of products available on the Polish market have demonstrated discrepancies between declared and measured monacolin K concentrations, although citrinin was not detected [36].

Finally, mechanistic interpretations remain speculative due to the absence of cholesterol absorption or synthesis biomarkers, and no sensitivity analyses were conducted to assess robustness to missing data. Therefore, the present findings should be interpreted as associative and hypothesis-generating, underscoring the need for prospective, sex-stratified studies with standardized metabolic and safety monitoring.

## 8. Conclusions

In this retrospective analysis of female patients undergoing SG, we demonstrated that while the surgery itself induced significant early weight loss and favorable metabolic changes, postoperative supplementation with monacolin K provided an additional benefit, particularly in stabilizing LDL-C levels, supporting HDL-C improvement, and enhancing total cholesterol reduction during the weight-stable phase. The combination of SG-driven weight loss and monacolin K may act in a complementary manner, contributing to more comprehensive lipid profile optimization. Our findings indicate that monacolin K supplementation was associated with favorable lipid profile changes in the studied cohort; however, these observations should be interpreted in the context of evolving regulatory and clinical guidance regarding the use of monacolin K-containing nutraceuticals The strengths of this study include a detailed longitudinal follow-up, a focus on both early and delayed supplementation timing, and the integration of mechanistic insights into cholesterol metabolism after SG. However, the retrospective design, inclusion of exclusively female patients, and lack of direct assessment of cholesterol absorption and synthesis markers limit the generalizability of these findings and warrant further confirmation in larger, prospective, sex-stratified studies.

## Figures and Tables

**Table 1:** Baseline characteristics of study participants across intervention and control groups: intervention groups G1, G2, and G3, based on the timing of postoperative monacolin K supplementation, as well as a control group (CG), which did not receive supplementation. Values are presented as mean ± standard deviation for age, percentages for comorbidities, and mean values for BMI.

	G1	G2	G3	CG	*p*
n	46	10	31	62	–
Age, years	38.7 ± 10.1	38.9 ± 4.7	37.3 ± 10.4	39.7 ± 10.0	0.76
Comorbidities, n (%)	37 (80.4)	9 (90.0)	18 (58.1)	40 (64.5)	0.06
BMI at baseline, kg/m^2^	42.0 (40.0–46.5)	39.8 (38.3–40.5)	43.3 (41.0–47.5)	42.0 (40.3–46.0)	0.27

Data presented as mean ± standard deviation or median (lower quartile; upper quartile). CG: control group without supplementation. G1: early postoperative monacolin K supplementation (supplementation within the first postoperative week). G2; initiated supplementation between months 3 and 9 postoperatively. G3: initiated supplementation between months 6 and 12.

**Table 2:** Lipid parameters in women undergoing SG with monacolin K supplementation (G1, G2, G3), and non-supplemented controls (CG). Values are presented as means with 95% confidence intervals at baseline (BO) and subsequent follow-up visits (T3 and T6 for G1 and CG; T6 and T9 for G2; T9 and T12 for G3). ANOVA results are reported for the effect of time (p_Time_), group (p_Group_), and group-by-time interaction (P_Int_). * *p* < 0.05; ** *p* < 0.01; ^#^ *p* < 0.001; *p* < 0.1 versus BO.

	G1			G2			G3			CG			ANOVA		
[mg/dL]	BO	T3	T6	BO	T6	T9	BO	T9	T12	BO	T3	T6	p_Time_	p_Group_	P_Int_
TC	213.1 (203.3–222.9)	205.2 (195.6–214.9)	199.7 ** (190.9–208.4)	214.9 (194.3–235.5)	200.7 (180.2–221.2)	192.4 * (173.8–211.0)	210.9 (199.1–222.7)	211.8 (200.0–223.6)	214.8 (203.9–225.7)	208.0 (199.4–216.6)	200.1 (187.8–212.3)	205.9 (198.1–213.6)	<0.05	0.54	0.16
HDL	51.4 (47.6–55.2)	48.3 (45.3–51.3)	55.5 * (52.1–58.9)	53.3 (45.4–61.2)	55.7 (49.5–61.9)	59.7 (52.1–67.3)	53.0 (48.5–57.5)	60.3 ^#^ (56.8–63.9)	64.7 ^#^ (60.4–68.9)	53.1 (49.8–56.4)	49.1 (44.6–53.5)	57.2 ^#^ (54.2–60.2)	<0.001	<0.001	<0.01
LDL-C	131.7 (120.8–142.6)	130.0 (118.0–141.9)	120.6 * (111.7–129.5)	128.9 (105.7–152.1)	121.9 (96.8–147.0)	114.1 (95.5–132.7)	128.7 (115.7–141.8)	126.6 (112.4–140.9)	126.0 (115.1–136.9)	99.9 (90.2–109.5)	93.0 (77.0–109.0)	121.8 * (114.0–129.5)	0.48	<0.01	<0.001
TG	151.9 (135.5–168.3)	129.8 ** (115.0–144.5)	115.7 ^#^ (100.2–131.2)	164.1 (129.0–199.2)	114.9 ** (83.8–146.0)	102.5 ** (69.5–135.5)	146.8 (126.9–166.8)	122.4 ** (104.7–140.1)	118.8 * (99.6–138.0)	148.1 (133.6–162.5)	132.1 (114.3–149.9)	128.4 * (114.5–142.3)	<0.001	0.88	0.41

Data presented as mean with 95% confidence interval.

**Table 3:** Percentage of excess weight loss (%EWL) and total body weight loss (%TBWL) across study groups G1, G2, G3 and CG at follow-up. Data are presented as mean values with 95% confidence intervals (CIs).

Δ_T2-BO_	G1	G2	G3	CG	*p*
% EWL	62.6 (55.6–59.6)	69.4 (52.3–86.5)	63.5 (54.4–72.7)	61.0 (55.1–66.9)	0.74
% TBWL	24.2 (21.6–26.8)	26.3 (21.7–31.0)	26.6 (23.5–29.7)	24.7 (22.6–26.8)	0.60

CG: control group without supplementation. G1: early postoperative monacolin K supplementation (supplementation within the first postoperative week). G2: initiated supplementation between months 3 and 9 postoperatively. G3: initiated supplementation between months 6 and 12.

## Data Availability

The data presented in this study are not publicly available due to ethical and privacy restrictions related to patient confidentiality. Aggregated data supporting the findings of this study are available from the corresponding author upon reasonable request.

## Supplementary Materials

The following supporting information can be downloaded at: https://www.mdpi.com/article/10.3390/nu18040647/s1, Figure S1: Changes in fasting glucose and HbA1c after sleeve gastrectomy in supplemented and non-supplemented groups; Table S1: Liver enzymes before and after monacolin K supplementation (G1).
