# Supplementary material for: Postoperative Monacolin K Supplementation and Lipid Profile After Sleeve Gastrectomy: A Retrospective Comparative Analysis in Women
Source: Nutrients. 2026 Feb 16;18(4):647. doi: 10.3390/nu18040647 (PMC12943251; doi:10.3390/nu18040647)
Supplement: Supplementary file 1 [file nutrients-18-00647-s001.zip › nutrients-4127919-supplementary.pdf]

Table S1. Liver enzyme levels before and after 6 months of monacolin K supplementation in the G1 supplementation group (n= 46).

Parameter	Before supplementation	After supplementation	Paired t-test (p)	Wilcoxon test (p)
**AST (U/L)**				
Mean ± SD	16.82 ± 3.03	15.18 ± 3.92	**0.049**	0.057
Median (IQR)	17.0 (2.0)	15.0 (3.0)		
**ALT (U/L)**				
Mean ± SD	15.09 ± 5.82	13.82 ± 5.31	0.051	**0.046**
Median (IQR)	13.0 (5.5)	12.0 (3.5)		

Data represent paired measurements obtained before initiation and after approximately 6 months of monacolin K supplementation. Values are presented as mean ± standard deviation and median with interquartile range (IQR). Paired t-tests and Wilcoxon signed-rank tests were used; Wilcoxon testing served as sensitivity analysis due to small sample size. All values remained within laboratory reference ranges.

Supplement

Fasting Glucose

Fasting glucose levels declined significantly across all study groups after SG. In the early supplementation group (G1), values decreased from 5.6 to 4.9 mmol/L ($p < 0.001$), corresponding to an absolute change of -0.65 mmol/L (95% CI: -0.91 to -0.38) and a relative reduction of -10.9% (95% CI: -15.0 to -6.8%). A similar improvement was observed in the control group (CG), with a decrease of -0.61 mmol/L (95% CI: -0.85 to -0.37; Δ% = -9.3). Among patients with delayed supplementation, fasting glucose dropped from 5.3 to 4.9 mmol/L in G2 (Δ = -0.43 mmol/L; 95% CI: -1.05 to 0.20), whereas in G3 the reduction was more pronounced, from 5.9 to 5.2 mmol/L ($p < 0.001$), representing an absolute change of -0.75 mmol/L (95% CI: -1.06 to -0.43) and a relative decrease of -12.5% (95% CI: -17.4 to -7.6%) (Fig. S1: A, B). Despite these consistent improvements across groups, neither group effects nor group-by-time interactions reached statistical significance.

A1c

A1c levels decreased consistently across all study groups. In the early supplementation group (G1), values declined from 5.9% to 5.6% ($p < 0.05$), with a delta of -0.24% (95% CI: -0.45 to -0.01; Δ% = -3.1%), comparable to the reduction

observed in the control group (CG), where the change was -0.21% (95% CI: -0.43 to 0.01%; Δ% = -3.1%). Among patients with delayed supplementation, A1c dropped from 6.2% to 5.6% in G2 (p < 0.05), corresponding to an absolute change of -0.57% (95% CI: -1.04 to -0.10%; Δ% = -8.5%), while G3 showed a more pronounced decline from 5.9% to 5.3% (p < 0.001), with Δ = -0.59% (95% CI: -0.86 to -0.31) and a relative reduction of -9.5% (95% CI: -13.7 to -5.3%). Despite these improvements, no significant group or interaction effects were observed, although a strong overall time effect was confirmed (p < 0.001) (Fig. S1: C, D).

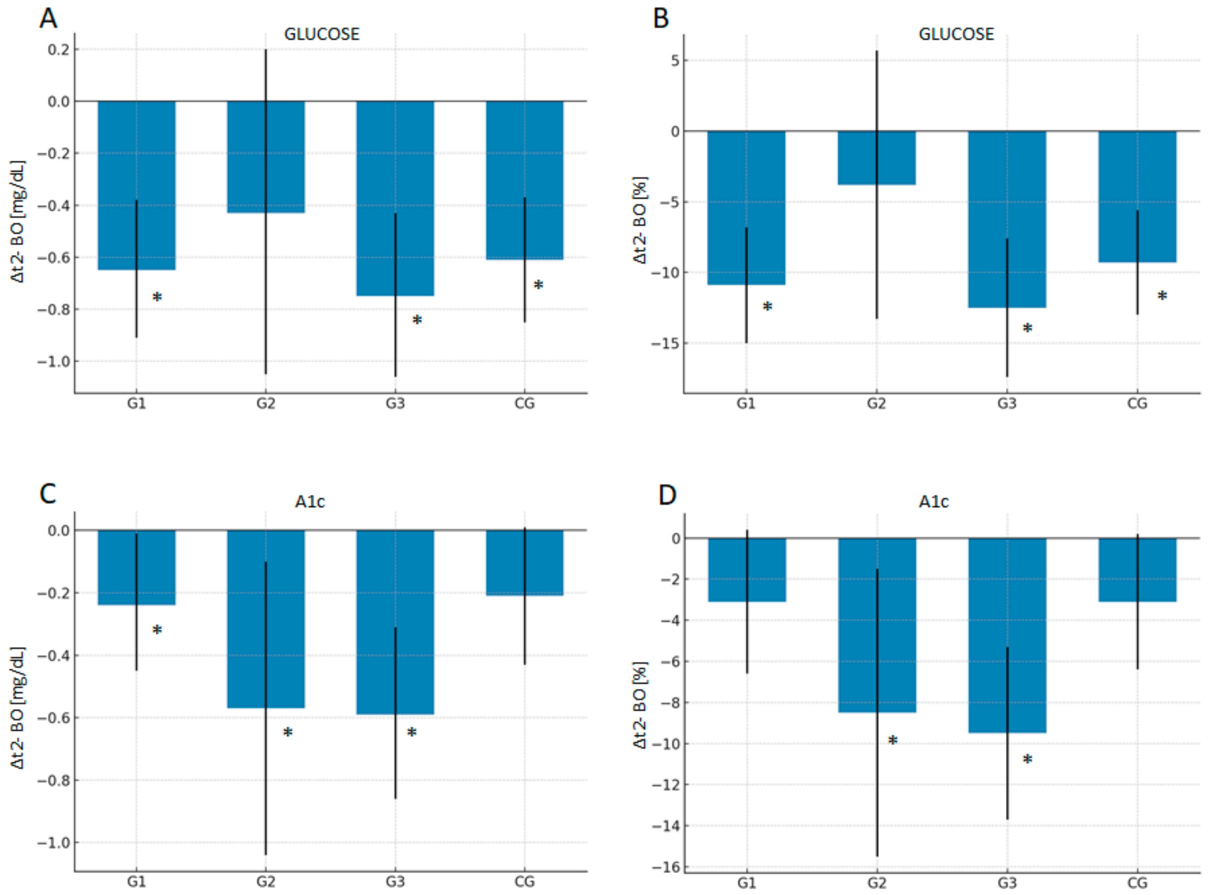

Figure S1. Absolute and relative changes in glucose and A1c from baseline (BO) to the second follow-up in groups G1, G2, G3, and CG (mean ± 95% CI). (A) Absolute changes in fasting glucose (ΔT2–BO, mg/dL). (B) Relative changes in fasting glucose (ΔT2–BO, %). (C) Absolute changes in A1c (ΔT2–BO, %). (D) Relative changes in A1c (ΔT2–BO, %). For G1 and CG, T2 corresponds to the 6-month follow-up, for G2 to the 9-month follow-up (T9), and for G3 to the 12-month follow-up (T12). * indicates significant differences between BO and the respective second follow-up ($p < 0.05$).

https://doi.org/10.3390/nu18040647
